# The History of a Human Anencephalus

**Published:** 1845-07

**Authors:** 


*The History of a Human Anencephalus.*—The following case confirms the existence of a system of excito-motory functions dependent on the spinal marrow, independently of the brain. A loaded cannon took off the head of the soldier who fired it; nothing was left of the face but the tongue and the lower jaw. The tongue was in violent convulsive motion, and the respiration deep, slow, and rattling. Both continued in this state for seven minutes and a half, when they became gradually slower and more rare, and ultimately ceased 15 minutes from the time of the accident. The whole brain (cerebrum and cerebellum) lay torn and scattered about; a few small pieces of the ossa parietalia were still hanging to torn pieces of the skin; the os basilare, with the foramen occipitale was still present, and showed the torn portion of the medulla oblongata.—*Ibid, from Dr. Schleifer in Oester. Medic. Wochenschr.*

